# Supplementary material for: Multi‐dataset identification of innovative feature genes and molecular mechanisms in keratoconus
Source: J Cell Mol Med. 2024 Sep 19;28(18):e70079. doi: 10.1111/jcmm.70079 (PMC11412914; doi:10.1111/jcmm.70079)
Supplement: Supplementary file 2 — Material S2. [file JCMM-28-e70079-s002.zip › 7.GSEA analysis/3.DBNDD1/KEGG_FC_EPSILON_RI_SIGNALING_PATHWAY.html]

Details for gene set KEGG\_FC\_EPSILON\_RI\_SIGNALING\_PATHWAY[GSEA]

|  || Dataset | DBNDD1.group.cls#h\_versus\_l |
| Phenotype | group.cls#h\_versus\_l |
| Upregulated in class | l |
| GeneSet | KEGG\_FC\_EPSILON\_RI\_SIGNALING\_PATHWAY |
| Enrichment Score (ES) | -0.37270436 |
| Normalized Enrichment Score (NES) | -1.3216169 |
| Nominal p-value | 0.09343936 |
| FDR q-value | 0.7828112 |
| FWER p-Value | 0.953 |
Table: GSEA Results Summary

  

Fig 1: Enrichment plot: KEGG\_FC\_EPSILON\_RI\_SIGNALING\_PATHWAY      
 Profile of the Running ES Score & Positions of GeneSet Members on the Rank Ordered List

  

| SYMBOL | TITLE | RANK IN GENE LIST | RANK METRIC SCORE | RUNNING ES | CORE ENRICHMENT || 1 | PIK3R5 | na | 35 | 0.708 | 0.0775 | No |
| 2 | RAC3 | na | 1824 | 0.312 | 0.0604 | No |
| 3 | IL3 | na | 3952 | 0.215 | 0.0229 | No |
| 4 | INPP5D | na | 4602 | 0.193 | 0.0256 | No |
| 5 | LCP2 | na | 5876 | 0.154 | 0.0059 | No |
| 6 | PLA2G2F | na | 6705 | 0.131 | -0.0034 | No |
| 7 | PIK3R1 | na | 6912 | 0.126 | 0.0046 | No |
| 8 | PLA2G10 | na | 7003 | 0.124 | 0.0158 | No |
| 9 | PLA2G2D | na | 8134 | 0.101 | -0.0056 | No |
| 10 | SOS1 | na | 8586 | 0.093 | -0.0084 | No |
| 11 | PLA2G12B | na | 9010 | 0.085 | -0.0111 | No |
| 12 | AKT2 | na | 9388 | 0.080 | -0.0131 | No |
| 13 | MAPK3 | na | 9865 | 0.073 | -0.0187 | No |
| 14 | MAPK10 | na | 10497 | 0.065 | -0.0298 | No |
| 15 | MAPK11 | na | 10512 | 0.064 | -0.0231 | No |
| 16 | FCER1G | na | 10592 | 0.063 | -0.0183 | No |
| 17 | VAV2 | na | 10635 | 0.063 | -0.0126 | No |
| 18 | MAP2K2 | na | 10931 | 0.059 | -0.0145 | No |
| 19 | MAPK9 | na | 11537 | 0.052 | -0.0263 | No |
| 20 | PDPK1 | na | 11670 | 0.050 | -0.0245 | No |
| 21 | PIK3R2 | na | 11699 | 0.050 | -0.0197 | No |
| 22 | MAPK13 | na | 11811 | 0.049 | -0.0175 | No |
| 23 | PLCG1 | na | 11955 | 0.048 | -0.0164 | No |
| 24 | SOS2 | na | 13580 | 0.033 | -0.0595 | No |
| 25 | PLA2G6 | na | 14048 | 0.030 | -0.0697 | No |
| 26 | KRAS | na | 14127 | 0.029 | -0.0688 | No |
| 27 | PIK3R3 | na | 14144 | 0.029 | -0.0660 | No |
| 28 | RAC1 | na | 14759 | 0.025 | -0.0810 | No |
| 29 | PLA2G4A | na | 15676 | 0.018 | -0.1055 | No |
| 30 | VAV3 | na | 16809 | 0.011 | -0.1370 | No |
| 31 | PIK3CA | na | 17073 | 0.009 | -0.1436 | No |
| 32 | MAPK14 | na | 17505 | 0.006 | -0.1553 | No |
| 33 | PLA2G2E | na | 17656 | 0.005 | -0.1590 | No |
| 34 | PLA2G12A | na | 18106 | 0.002 | -0.1718 | No |
| 35 | PIK3CB | na | 18338 | 0.000 | -0.1784 | No |
| 36 | MAP2K4 | na | 18667 | -0.002 | -0.1877 | No |
| 37 | HRAS | na | 18984 | -0.004 | -0.1963 | No |
| 38 | RAF1 | na | 19005 | -0.005 | -0.1964 | No |
| 39 | AKT1 | na | 19066 | -0.005 | -0.1975 | No |
| 40 | PLCG2 | na | 19340 | -0.007 | -0.2046 | No |
| 41 | SYK | na | 19415 | -0.008 | -0.2059 | No |
| 42 | PLA2G4E | na | 19468 | -0.008 | -0.2065 | No |
| 43 | MAP2K7 | na | 19930 | -0.012 | -0.2184 | No |
| 44 | PRKCD | na | 20754 | -0.020 | -0.2400 | No |
| 45 | RAC2 | na | 20865 | -0.021 | -0.2408 | No |
| 46 | PRKCA | na | 21412 | -0.026 | -0.2537 | No |
| 47 | GRB2 | na | 22294 | -0.035 | -0.2753 | No |
| 48 | PLA2G2C | na | 22365 | -0.036 | -0.2733 | No |
| 49 | MAP2K3 | na | 22519 | -0.037 | -0.2736 | No |
| 50 | MAPK8 | na | 22620 | -0.039 | -0.2722 | No |
| 51 | MAPK1 | na | 22705 | -0.040 | -0.2702 | No |
| 52 | JMJD7-PLA2G4B | na | 23109 | -0.044 | -0.2769 | No |
| 53 | PIK3CD | na | 24971 | -0.070 | -0.3228 | No |
| 54 | MAPK12 | na | 25666 | -0.081 | -0.3338 | No |
| 55 | NRAS | na | 27014 | -0.108 | -0.3607 | Yes |
| 56 | TNF | na | 27164 | -0.111 | -0.3527 | Yes |
| 57 | PIK3CG | na | 27455 | -0.118 | -0.3480 | Yes |
| 58 | IL13 | na | 28093 | -0.133 | -0.3516 | Yes |
| 59 | LYN | na | 28223 | -0.136 | -0.3403 | Yes |
| 60 | BTK | na | 28336 | -0.139 | -0.3281 | Yes |
| 61 | PLA2G3 | na | 28607 | -0.146 | -0.3196 | Yes |
| 62 | IL5 | na | 28795 | -0.151 | -0.3083 | Yes |
| 63 | VAV1 | na | 28859 | -0.153 | -0.2931 | Yes |
| 64 | PLA2G5 | na | 29074 | -0.159 | -0.2817 | Yes |
| 65 | MAP2K1 | na | 29246 | -0.164 | -0.2684 | Yes |
| 66 | MAP2K6 | na | 29536 | -0.173 | -0.2575 | Yes |
| 67 | LAT | na | 29791 | -0.182 | -0.2447 | Yes |
| 68 | CSF2 | na | 30210 | -0.195 | -0.2351 | Yes |
| 69 | FYN | na | 31175 | -0.227 | -0.2377 | Yes |
| 70 | FCER1A | na | 31450 | -0.238 | -0.2192 | Yes |
| 71 | PLA2G1B | na | 31847 | -0.256 | -0.2023 | Yes |
| 72 | GAB2 | na | 32145 | -0.272 | -0.1807 | Yes |
| 73 | MS4A2 | na | 32191 | -0.276 | -0.1514 | Yes |
| 74 | PRKCE | na | 32256 | -0.280 | -0.1222 | Yes |
| 75 | AKT3 | na | 33656 | -0.385 | -0.1200 | Yes |
| 76 | IL4 | na | 33666 | -0.386 | -0.0774 | Yes |
| 77 | PRKCB | na | 34153 | -0.452 | -0.0413 | Yes |
| 78 | PLA2G2A | na | 34424 | -0.522 | 0.0088 | Yes |
Table: GSEA details [plain text format]

  

Fig 2: KEGG\_FC\_EPSILON\_RI\_SIGNALING\_PATHWAY      
 Blue-Pink O' Gram in the Space of the Analyzed GeneSet

  

Fig 3: KEGG\_FC\_EPSILON\_RI\_SIGNALING\_PATHWAY: Random ES distribution      
 Gene set null distribution of ES for **KEGG\_FC\_EPSILON\_RI\_SIGNALING\_PATHWAY**
